# Supplementary material for: Whole Transcriptome Analysis Identifies Platycodin D-Mediated RNA Regulatory Network in Non–Small-Cell Lung Cancer
Source: Cells. 2022 Aug 1;11(15):2360. doi: 10.3390/cells11152360 (PMC9367903; doi:10.3390/cells11152360)
Supplement: Supplementary file 1 [file cells-11-02360-s001.zip › Supplementary file.pdf]

SUPPLEMENTARY INFORMATION CAPTION:

Supplementary Table S1

Divergent primers for DE-circRNAs

Supplementary Table S2

Convergent primers for DE-circRNAs

Supplementary Table S3

Reads of transcripts with annotation and replicates

Supplementary Figure S1

Heatmap of DE-mRNAs between PD treated A549 cells and the control

Supplementary Figure S2

DNA gel electrophoresis of hsa_circ_0001326, hsa_circ_0005870 and hsa_circ_0006990 amplification products

Supplementary Figure S3

Relative expression of DE-lncRNAs in PD treated A549 cells and the control

Supplementary Figure S4

The effect of PD on A549 cell 3D culture

Supplementary Data

Table S1. Divergent primers for DE-circRNAs

Name	F-primer (5’-3’)	R-primer (5’-3’)
hsa_circ_0001326	GTTGGTGAAAAGACCAAGGAGAG	GCTTCCTCAAACACAGACTCCTC
hsa_circ_0001946	CAGTGTCTGCAATATCCAGGGTT	TGGAAGACCTTGAGATTATTGGAA
hsa_circ_0005870	AGTGGGATCCTCCTACTTGGG	ACTTGGCTGGGCATAACTCTTC
hsa_circ_0006702	TTTGAGACTGAAATGGCAGATCA	TCAGCCAATTTCCAAATCGTT
hsa_circ_0006990	TGGAAAGAGGCAAAACCTGATG	CTACATCTGTGAAGGGGCCAAT

Table S2. Convergent primers for DE-circRNAs

Name	F-primer (5’-3’)	R-primer (5’-3’)
hsa_circ_0001326	GTGCTCTTTTGGATGGAGAACA	CGGTGGTCCCAGTAGATAAGC
hsa_circ_0001946	CTTCCAACGTCTCCAGTGTG	AGTCGCTGGAAGACCCG
hsa_circ_0005870	GGGAAAGCCCAGGAGATGATG	ACTATTGTAGTCACTGCTGCGG
hsa_circ_0006702	AAGCCCCAACATTACAGACCG	GTCCACCATGTTCTCCACTCG
hsa_circ_0006990	TGGAAGCTGTGTGGAAAGAGG	GCGAGGTGCTGTAGTCTTCAC

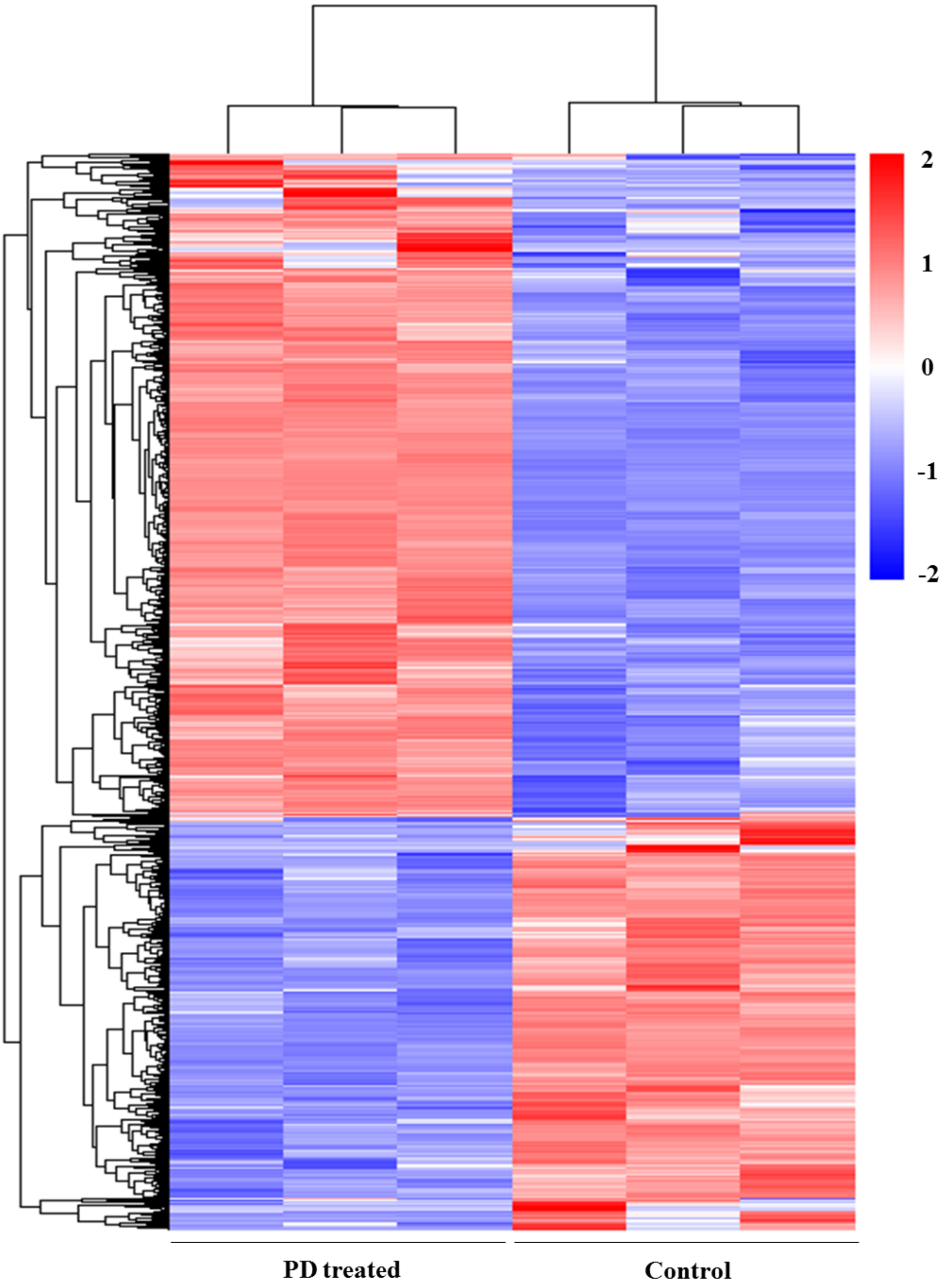

Figure S1. Heatmap of DE-mRNAs between PD treated A549 cells and the control. Red indicates upregulation, and blue indicates downregulation (p-adj < 0.05).

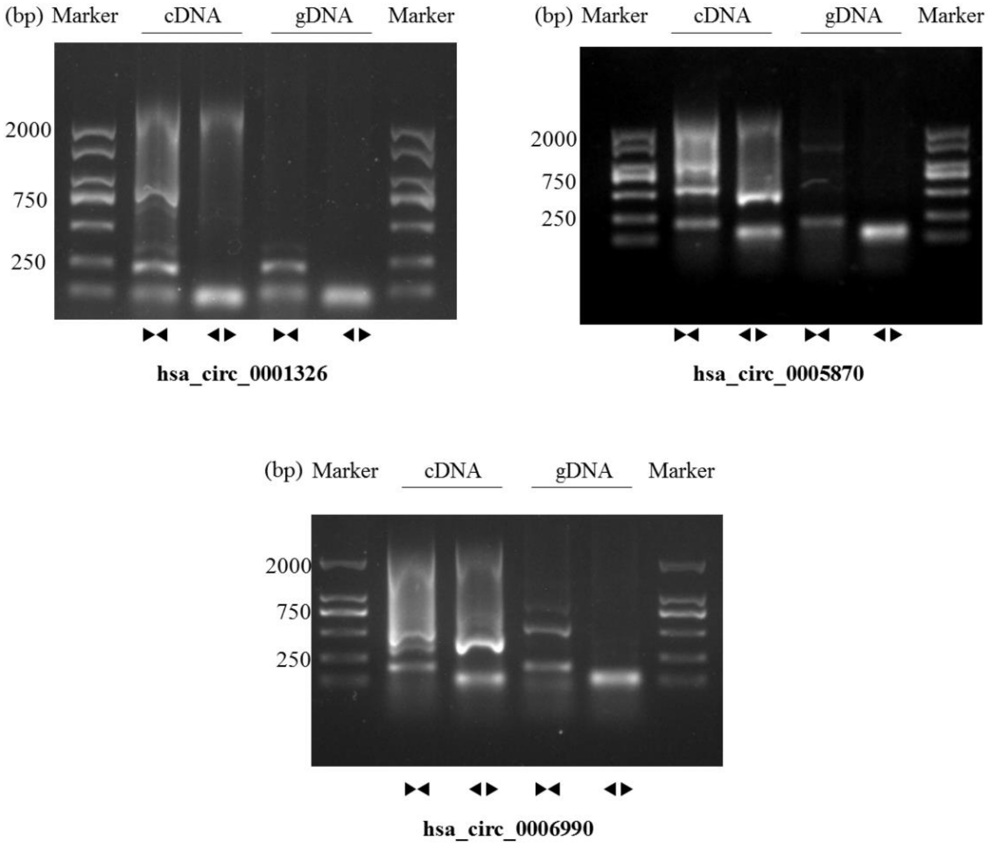

Figure S2. DNA gel electrophoresis of hsa_circ_0001326, hsa_circ_0005870 and hsa_circ_0006990 amplification products.

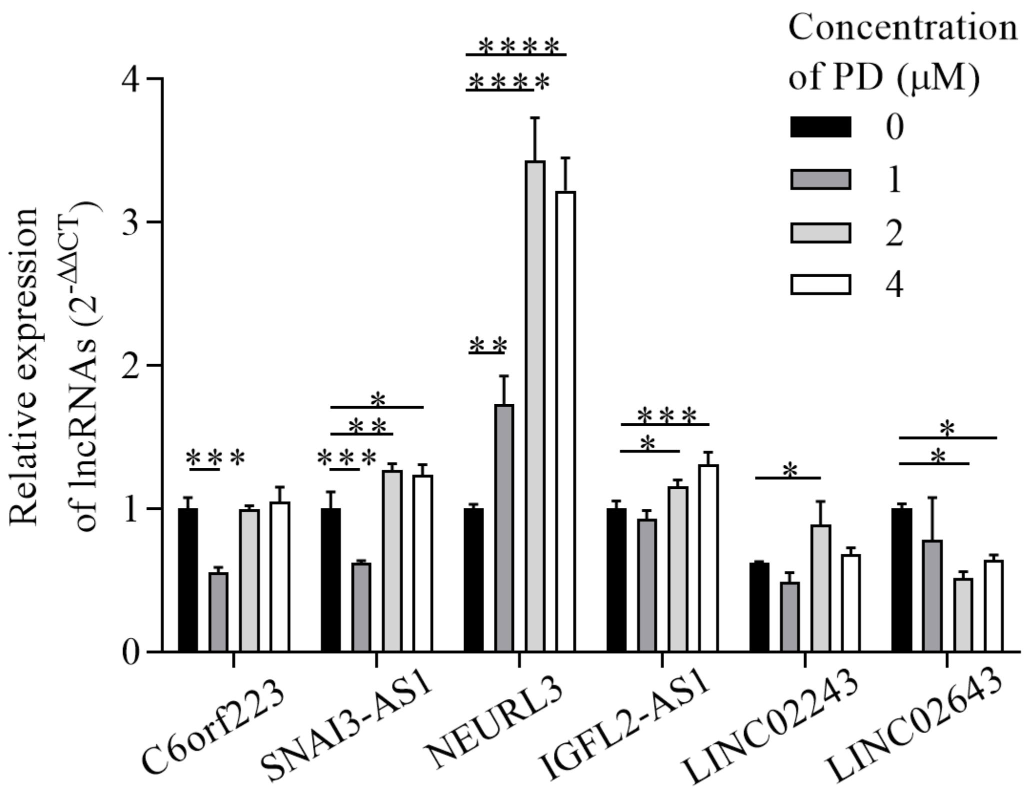

Figure S3. Relative expression of DE-lncRNAs in PD treated A549 cells and the control.

U6 is used as internal control. * P<0.05; ** P<0.01; *** P<0.001; **** P<0.0001

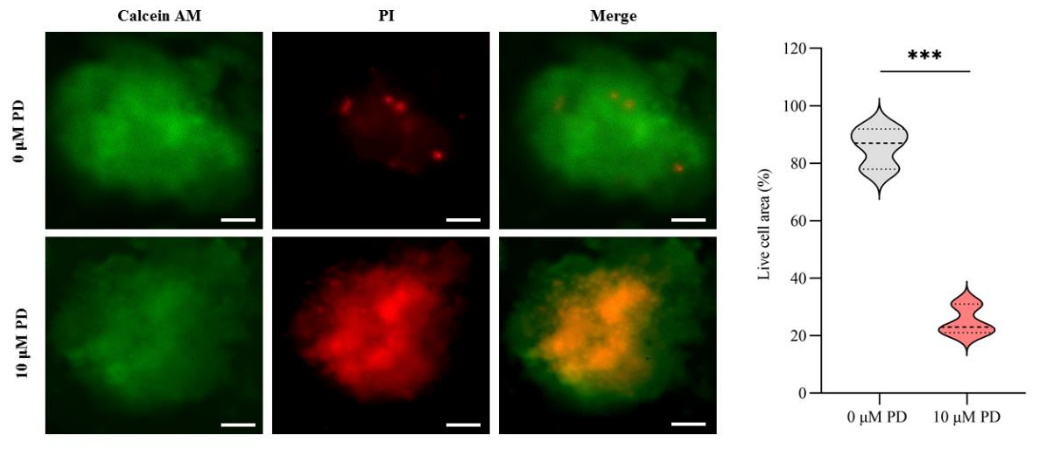

Figure S4. The effect of PD on A549 cell 3D culture.

Live cells are stained by Calcein-AM (green color), and dead cells are stained by PI (red color). Scale bar: 100 μm. *** P<0.001

Supplementary Data

Hsa_circ_0001946 Sanger sequencing data (5'-3')
>AGTCGCTGGAAGACCCGGAGTTGTTGGAAGACCTTGACACAGGTGCCAT
CGGAAACCCTGGATATTGCAGACACTGGAAGACCTGAATGTCAGAAGATC
AGCACACTGGAGACGTTGGAAGACATGGATATTGAGCCAGTTGATGGAAG
ACTGGGTAGTTGTTGGAAGACATGAAGATGCTGGAAGACACAGAGATGCT
GGAAGACGTGGAGATGTTGGAAGACGAGCAGATGCTGGAAGCCCTGGAG
ATGCTGGAAGACCTGGAGATATAGGAAGACATGGATTTGTTGGAAGACGTA
GCTTAGTTGGAAGACATATATTTCTGGAAGACGTGGATTTCTGGAAGAC
ATGGCTTGGTTGGAAGACGTGGATTTCTGGAAGACGTACCTTTGTTGGAA
GACATACCTTTGTTGGAAGACGTACCTTTGTTGGAAGACGTACCTTTGTTG
GAAGACACAAGTAGGCTGGAAGACATTAATTTGATGGAAGACATGGCCTT
GTTGGAAGACGTGGATTTGCTGGAAGACACGGATTTCCTGGAAGACCTGG
ATTTTTCGGAAGCTATGGATTTGAGGGAAGACAAGGATTTTCTGGAAGACA
TGGATAGTCTGGAAGACATGGCTTTGTTGGAAGACGTGGACTTGCTGGAA
GACACGGATTTCCTGGAAGACCCGGATTTTTTGGAAGCTATAGATTTAAGG
GAAGACAAGGATTTTCTGGAAGACATGGATAGTCTGGAAGACCTGGAGGC
CATTGGAAGATGTGGATTTTCTGGAAGACATGGCTTTTTTGGAAGACGTAG
ATTTTCAGGAAGACCCAAATTATCCGGAAGACTTGGATTGTTGGGAAGA-
CGTGGATTTTCTGGAAGACTGGGAGGTTACTGGAAGACATGGATTTTCTGG
AAGACATGGATTTTCTGGAAGACGTGGATCTTCAGGAAGACATATATTGGC
TGGAAGACCTGGATTTTTTCCGGAAGATGTGGATTGACTGGAAGACCTGGA
TTTGGTGGAAGACGTAGATTTTTCTGGAAGACACTGACTGACTGGAAGAC
CTGGATTTCTTTCTGGAAGGCACTGATTGACTGGAAGACCTGGATTTCTTTC
TGGAAGACACTGATTGACTGGAAGATCTAGATTTTTCTGGAAGAACTAGAT
TTACTGGAAGACTTGGATTTGGTGGAAGACACAGATTTTTCTGGAAGACAT
GGATTAGCTGGAAGATCTGTATTTGATGGAAGACCTTGAAATTATTGGAAG
ACATGGATTTCCTGGAAGACGTGGATTTTCCTGGAAGATCTGGATTTGGTG
GAAGACCAGTAATTGCTGGAAGACTGGATTTGCTGGAAGACTTGATTTACT
GGAAGACTTGGAGCTTCTTGGAAGACATGGATTGTCCGGAAGACATGGATT
GTCTGGAAGATGTGGATTTTCTGGAAGCTCAGGATTATCTGGAAGACCTTG
AGATTATTGGAAGACTTGAAGTCGCTGGAAGACCCGGAGTTGTTGGAAGA
CCTTGACACAGGTGCCATCGGAAACCCTGGATATTGCAGACACTGGAAGA
CCTGAATGTCAGAAGATCAGCACACTGGAGACGTTGGAAG

Hsa_circ_0006702 Sanger sequencing data (5'-3')
>TTACACTGCAATCGCGTGTCGCCCTTAAGCCCCAACATTACAGACCGGTG
GATCCTGTCCTTCATGCAGTCTCTCATTGGCTTCTTTGAGACTGAAATGGCA
GATCAGACACTCCTCTAATTTACAAAGCAGTGCCCAGCTGGTTTGTGCGAG
TGGAGAACATGGTGGACCAGCTCCTAAGGAACAATGACCTGTGCTACTGG
TGAGCAGGTCCCAGAGTTGGTACGAGAAAAACGATTTGGAAATTGGCTGA
AAGATGCACGTGACTGGACAATTTCCAGAAACAGATACTGGGGCACCCCC

ATCCCACTGTGGGTCAGCGATGACTTTGAGGAGGTGGTATGCATTGGGTCA
GTGGCGGAACTTGAAGAACTGTCAGGAGCAAAGATCTCAGATCTCCACAG
AGAGAGTGTTGACCACCTGACCATTCCTTCACGCTGTGGGAAGGGATCCTT
GCACCGCATCTCTGAAGTGTTTGACTGTTGGTTTGAGAGTGGCAGCATGCC
CTATGCTCAGGTTCATTACCCGTTTGAAAACAAGAGGGAGTTTGAGGATGC
TTTTCCTGCAGATTTCATTGCCGAGGGCATCGACCAAACCAGAGGATGGTT
TTATACCCTGCTGGTGCTGGCCACGGCCCTCTTTGGACAACCGCCTTTCAA
GAACGTAATTGTGAATGGGCTTGTCCTGGCAAGTGATGGCCAAAAAATGAG
CAAACGGAAAAAGAATTATCCAGATCCAGTTTCCATCATCCAGAAGTATGG
TGCTGATGCCCTCAGATTATATCTGATTAACTCCCCTGTGGTGAGAGCAGAA
AACCTCCGCTTTAAAGAAGAGGGTGTGCGGGACGTCCTTAAGGATGTACTG
CTCCCATGGTACAATGCCTATCGCTTCTTAATCCAGAACGTTCTGAGGCTCC
AGAAGGAGGAAGAAATAGAATTTCTCTACAATGAGAACACGGTTAGAGAA
AGCCCCAACATTACAGACCGGTGGATCCTGTCCTTCATGCAGTCTCTCATTG
GCTTCTTTGAGACTGAAATGGCAGATCAGACACTCCTCTAATTTACAAAGC
AGTGCCCAGCTGGTTTGTGCGAGTGGAGAACATGGTGGACAAGGGCGACA
CGCGAT
